# Morphological 3D Analysis of PLGA/Chitosan Blend Polymer Scaffolds and Their Impregnation with Olive Pruning Residues via Supercritical CO_2_

**DOI:** 10.3390/polym16111451

**Published:** 2024-05-21

**Authors:** Ignacio García-Casas, Diego Valor, Hafsa Elayoubi, Antonio Montes, Clara Pereyra

**Affiliations:** Department of Chemical Engineering and Food Technology, Faculty of Sciences, International Excellence, Agrifood Campus (CeiA3), University of Cádiz, 11510 Puerto Real, Spain; ignacio.casas@uca.es (I.G.-C.); elayoubihafsa@gmail.com (H.E.); clara.pereyra@uca.es (C.P.)

**Keywords:** scaffolds, polymers, 3D analysis, DragonFly, PLGA, chitosan, olive pruning, CO_2_

## Abstract

Natural extracts, such as those from the residues of the *Olea europaea* industry, offer an opportunity for use due to their richness in antioxidant compounds. These compounds can be incorporated into porous polymeric devices with huge potential for tissue engineering such as bone, cardiovascular, osteogenesis, or neural applications using supercritical CO_2_. For this purpose, polymeric scaffolds of biodegradable poly(lactic-co-glycolic acid) (PLGA) and chitosan, generated in situ by foaming, were employed for the supercritical impregnation of ethanolic olive leaf extract (OLE). The influence of the presence of chitosan on porosity and interconnectivity in the scaffolds, both with and without impregnated extract, was studied. The scaffolds have been characterized by X-ray computed microtomography, scanning electron microscope, measurements of impregnated load, and antioxidant capacity. The expansion factor decreased as the chitosan content rose, which also occurred when OLE was used. Pore diameters varied, reducing from 0.19 mm in pure PLGA to 0.11 mm in the two experiments with the highest chitosan levels. The connectivity was analyzed, showing that in most instances, adding chitosan doubled the average number of connections, increasing it by a factor of 2.5. An experiment was also conducted to investigate the influence of key factors in the impregnation of the extract, such as pressure (10–30 MPa), temperature (308–328 K), and polymer ratio (1:1–9:1 PLGA/chitosan). Increased pressure facilitated increased OLE loading. The scaffolds were evaluated for antioxidant activity and demonstrated substantial oxidation inhibition (up to 82.5% under optimal conditions) and remarkable potential to combat oxidative stress-induced pathologies.

## 1. Introduction

According to the International Olive Council, Spain is considered the world’s largest producer of olive oil, with a production of 1,251,300 tons in 2018, representing approximately 35% of global production [1]. The olive industry produces not only the prized oil as its main product but also liquid by-products such as olive mill wastewater and solid by-products such as olive pomace, leaves, and branches. Therefore, the valorization of these residues, which are generated in large quantities, has become a necessity to improve the profitability of the olive sector [2]. Nowadays, thanks to the development of technology and improved knowledge, the uses of olive leaves have expanded and diversified. In fact, these leaves are used for the extraction of interesting compounds such as mannitol, sterols, fatty alcohols, and phenolic compounds, mainly oleuropein, flavonoids, and triterpenic compounds [3]. Rubel et al. [4] found that the antioxidant and antimicrobial properties of olive leaves prolonged the shelf life of lamb meatballs. Antioxidant compounds play a crucial role in preserving foods and protecting organisms from free radical damage. Several studies have demonstrated the antioxidant activity of olive leaves, largely due to the presence of oleuropein and hydroxytyrosol, both of which act as potent free radical scavengers [5]. The antimicrobial activity of olive leaf extract against bacteria such as *E. coli*, *B. cereus*, *S. aureus*, *P. aeruginosa*, *C. albicans*, and others has been extensively studied [5,6].

When it comes to obtaining bioactive compounds present in low concentrations, traditional extraction techniques such as solid-phase extraction, solid-phase microextraction, and liquid–liquid extraction are not the most suitable options [7,8]. These techniques require long extraction times and involve the use of large amounts of sample and organic solvents, which are expensive and difficult to obtain, as well as having a negative impact on the environment and human health. Another major drawback of traditional extraction techniques is that the extracts obtained often require concentration and purification before analysis [9]. As a solution to the drawbacks associated with traditional extraction techniques, supercritical fluids (SCFs) are presented as an alternative solvent for the extraction of bioactive compounds. The physicochemical properties of supercritical fluids, such as high diffusivity, low viscosity, and high density, favor their penetration into solid matrices and the solubilization of different solutes. Carbon dioxide (CO_2_) is the most widely used supercritical fluid due to its easily achievable critical values, with a critical temperature of 304 K and critical pressure of 7.37 MPa. Additionally, CO_2_ is economical, inert, non-flammable, readily accessible, and environmentally friendly. Moreover, it can be easily separated from other process components through depressurization since it exists in a gaseous state at room temperature [10]. Enhanced solvent extraction (ESE) employs high concentrations of CO_2_ in combination with solvents at high pressure. This technique offers benefits such as improved mass transfer, reduced solvent consumption, and elimination of concentration steps that may degrade bioactive compounds [11]. Using supercritical CO_2_ as a solvent, extraction has been studied in numerous plants, such as Curcuma longa (10–35 MPa and 313–333 K) [12], rosemary leaves and flowers (45 MPa and 343 K) [13], eucalyptus leaves (10–50 MPa and 313–353 K) [14], and *Cannabis sativa* (17–34 MPa and 323 K) [15]. In particular, olive derivatives have also been extracted with supercritical CO_2_, including phytocompounds from olive pomace [16], olive leaves [17], and the extraction of lyophilized olive mill wastewater [18].

In the field of tissue engineering, numerous studies have emphasized the importance of using polymers that mimic the extracellular matrix, exhibiting characteristics such as interconnected porosity, permeability, and mechanical strength similar to those of living tissue [19]. These polymers, known as “scaffolds”, were initially designed as supports for repairing various tissues. There are various techniques for forming these scaffolds, most of which involve the polymerization of the monomer in solution in the presence of an oxidant and other non-conductive polymers, followed by the deposition of the mixture into molds and a subsequent freezing or drying process. However, these techniques pose significant challenges, such as the difficulty in completely removing the organic solvents used in the initial solutions. Additionally, many of these techniques require high temperatures that are not compatible with the inclusion of active principles, as well as long processing times that can cause stratification of substances within the scaffold, reducing its therapeutic efficacy. Furthermore, various studies indicate that conventional methods are not efficient enough to produce polymeric matrices with a similar or identical morphology, with uniformly sized pores and high interconnectivity between them [20]. For all these reasons, in recent decades, the use of supercritical fluids (SCFs) has been explored for obtaining polymeric scaffolds. Supercritical carbon dioxide (scCO_2_) acts as a solvent carrier and swelling agent for the porous matrix, ensuring the success of impregnation. Additionally, the moderate temperature and pressure conditions of scCO_2_ allow processes to be developed without degradation of heat-sensitive substances. The interaction between scCO_2_, the active substance, and the porous matrix is influenced by factors such as pressure, temperature, solubility, and absorption capacity of the polymer [21].

Biocompatible polymers such as poly(lactic-co-glycolic acid) (PLGA) and chitosan (Ch) are highly attractive and versatile. PLGA is a biodegradable polymer widely used as a matrix for encapsulating bioactive compounds, including potent antitumor agents such as doxorubicin and paclitaxel [22]. Additionally, PLGA is FDA-approved for use in contact with biological fluids, making it a reliable option. Its applications in the medical field are diverse, ranging from wound closure and surgical sutures to release carriers, tissue engineering scaffolds, and various types of implants. The use of scCO_2_ to fabricate PLGA scaffolds has been reported in several studies. Wang et al. [23] reported the fabrication of a multi-modal cell structure of PLGA using scCO_2_, Lee et al. [24] produced microcellular foaming of PLGA polymer that exhibits micrometer-sized pores (~50 μm) and shows great potential for new surgical implants for controlled release of paclitaxel, and Alvarez et al. [25] produced PLGA foams using CO_2_ at high pressure but below supercritical conditions. Other authors have mixed the formation of PLGA foams with other polymers or compounds such as polycaprolactone (PCL) [26,27], poly(3,4-etilendioxitiofeno) (PEDOT) [28], bioglass particles/poly(lactide-co-glycolide) (BG/PLGA) [29], Sr/Zn n-HAp and PLGA [30], or (PLGA)/superparamagnetic iron oxide nanoparticles (SPIONs) [23,31,32]. Chitosan (Ch), a biodegradable polysaccharide extracted from crustaceans, has been shown to be safe and non-toxic in both animals and humans. Due to its excellent biocompatibility, chitosan has been widely used in pharmaceutical research and industry as a carrier for drug delivery or as part of biocomposites [33]. In the study by Nie et al. [34], porous foams composed of PLGA/chitosan were manufactured using a combination of spray drying and foaming techniques with supercritical CO_2_. The effects of chitosan incorporation on certain factors were then investigated. The results of this study indicate that PLGA/Ch compounds show potential success in genetic engineering. One observed effect of chitosan incorporation is the alteration of the electrical properties of the scaffolds. Initially, the scaffold made solely of PLGA had a negative charge. However, with the incorporation of chitosan, the charge properties changed to be less negative or even positive. This change in charge may play a role in enhancing subsequent cell adhesion and viability. It was also observed that by increasing the chitosan content to 10% in the PLGA/Ch mixture, the foam structure acquired a flower-like appearance, with all pores interconnected and no apparent walls between them. The results of this study suggest that the inclusion of chitosan in the PLGA matrix has beneficial effects on scaffold properties, making it potentially more suitable for gene delivery applications.

On the other hand, the impregnation process involves embedding bioactive compounds into porous matrices. Traditional impregnation methods typically entail immersing the matrix in a solvent containing the desired compounds. However, these techniques present challenges such as the use of potentially toxic organic solvents, extended operation times, low specificity, and low yields [35]. Hence, more efficient impregnation methodologies based on the utilization of supercritical fluids (SCFs) have been developed, offering significant advantages. Supercritical CO_2_ is employed for impregnation with SCFs. For impregnation to take place, CO_2_ must come into contact with the solvent containing the compounds to be impregnated. During the impregnation process, two equilibria occur: the first between CO_2_ and the solvent of the extract, allowing the extract compounds to dissolve in CO_2_, and the second between the compounds dissolved in CO_2_ and the matrix onto which they will be deposited [36]. CO_2_ serves as an intermediary. It is crucial to identify the appropriate operating conditions to achieve satisfactory impregnation, as different compounds may exhibit varied behaviors, and certain conditions may be beneficial for some compounds but detrimental to others. Some studies of supercritical impregnation in PLGA scaffolds included mango leaves [28], bioactive lipids [37], or dexamethasone [38]. Braga et al. managed to impregnate ophthalmic drugs into chitosan [35].

This study consists of two distinct phases. First, it aims to elucidate the effect of incorporating a certain percentage of chitosan on the structural properties of PLGA scaffolds, including parameters such as pore count, interconnectivity, and porosity. This investigation will also include a comparative analysis with scaffolds prepared under identical conditions but supplemented with olive pruning waste extract. The second phase of the study focuses on an experimental design aimed at determining the optimal impregnation conditions. This investigation involves the systematic study of key parameters inherent to a process using supercritical carbon dioxide (scCO_2_), in particular pressure and temperature. In addition, the study introduces the variable of PLGA/Ch ratio to evaluate its influence on the impregnation efficiency.

## 2. Materials and Methods

### 2.1. Materials

Poly(lactic-co-glycolic acid) (PLGA) with a lactide:glycolide ratio of 75:25 and a molecular weight range of 76,000–115,000, DPPH (2,2-diphenyl-1-picrylhydrazyl), chitosan (medium molecular weight), gallic acid, and Folin–Ciocâlteu reagent (FCR) were acquired from Sigma–Aldrich (Madrid, Spain). Absolute ethanol was obtained from PanReac AppliChem (Barcelona, Spain). CO_2_ with a purity of 99.8% was supplied by Linde (Barcelona, Spain).

Olive pruning waste was gathered by the “Olivarera San José de Lora de Estepa Cooperative” in Seville, Spain. The waste was dried in an oven at 40 °C for one day. Subsequently, it was ground in an electric grinder for 1.5 min to prevent thermal degradation and then sieved to achieve an average particle diameter of approximately 1 mm. The processed material was stored at room temperature.

### 2.2. Enhanced Solvent Extraction from Olive Pruning Waste

**Figure 1:**
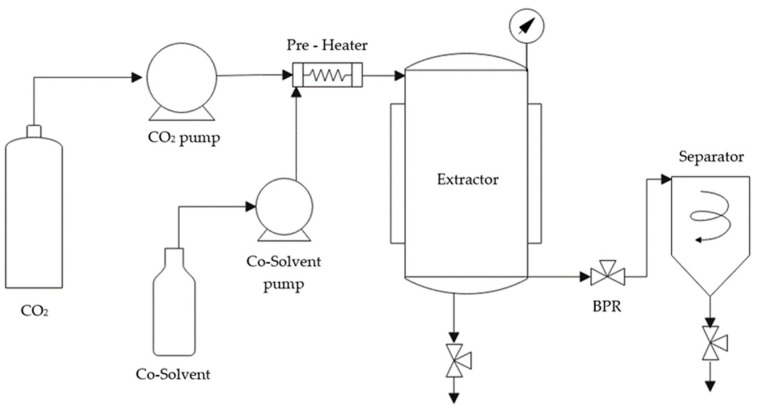
Schematic of ESE plant.

The extraction of olive pruning waste was produced using the enhanced solvent extraction (ESE) method explained by Cejudo et al. [39]. In this process 120 g of olive pruning was used over a 24 h period in an SF100 pilot plant (Figure 1) developed by Thar Technologies (Pittsburgh, PA, USA). The extraction procedure involved using 650 mL of ethanol as a co-solvent, conducted at a pressure of 120 bar, a temperature of 80 °C, and a flow rate of 10 g ethanol per minute. The ESE was carried out with a 50% CO_2_–50% ethanol ratio with a final concentration of 97.5 mg dry weight/mL ethanol.

### 2.3. Foaming and Impregnation Supercritical Process

**Figure 2:**
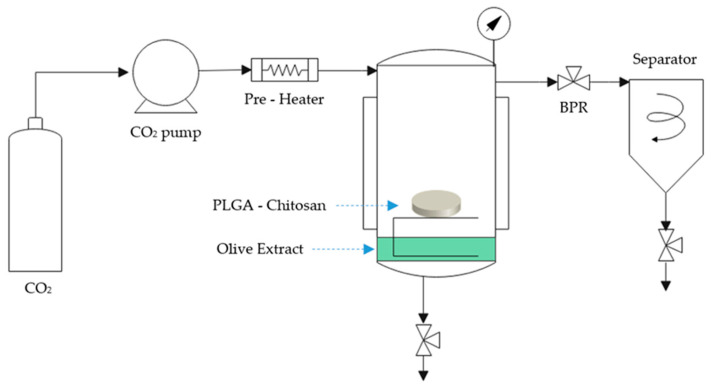
Schematic of Foaming and Impregnation plant.

A pilot plant developed by Thar Technologies, as illustrated in Figure 2, was utilized for foaming PLGA and PLGA/Ch and its previous morphological analysis, and then for foaming and impregnation in a one-step process to impregnate olive pruning waste into PLGA/Ch blend polymers scaffolds. This plant comprised a CO_2_ bottle, a chiller to cool and maintain CO_2_ in a liquid state before entering the high-pressure pump, and a heat exchanger to raise the CO_2_ temperature prior to its introduction into a high-pressure impregnation cell. Within this cell, 15 mL of ethanolic extract with a concentration of 20 mg/mL and a porous metal basket with a pore size of 10 μm, containing a 30 mg PLGA/Ch disk created by a compression machine, were introduced into the foaming vessel. CO_2_ was pumped into the vessel until the desired foaming/impregnation conditions were achieved. The polymer mixture was subsequently retained in the vessel. The polymer was allowed to come into contact with CO_2_ for a set amount of time to enable it to penetrate and cause plasticization. The system was then depressurized at a controlled rate using the automatic back-pressure regulator valve, resulting in foaming and impregnation of the final polymeric structure. This batch impregnation process was conducted ensuring no direct contact between the extract and the polymeric matrix.

Firstly, a morphological analysis of the effect of adding chitosan to PLGA on scaffold formation was studied. To this end, the effect of the PLGA/Ch ratio in weight was studied at 1:1, 7:3, and 9:1, and the morphological effect of olive extract impregnation on the same PLGA/Ch ratios was also studied (Table 1). For this purpose, the conditions of pressure (10 MPa), temperature (308 K), depressurization rate (10 MPa/min), and scCO_2_ flow (20 mg/min) were set.

In a further study, a mixed level 3 × 2 × 2 experiment with two center points was designed by Statgraphics Centurion XIX (version 19.6.03) software to study the effects of three independent variables—pressure (ranging from 10 to 30 MPa), temperature (308 to 328 K), and ratio of PLGA/Ch in weight (1:01–7:03–9:01)—on the impregnation process. The foaming and impregnation time and depressurization rate were fixed to 2 h and 10 MPa/min. The amount of PLGA/Ch in the disk (30 mg) and volume of extract (15 mL) were also kept constant throughout the process. A summary of the conducted experiments is provided in Table 2.

### 2.4. Tomography Analysis: Porosity, Connectivity, and Expansion Degree

X-ray computed microtomography (µ-CT) scans were performed on a Zeiss Xradia 610 Versa (Oberkochen, Alemania) to study the influence of PLGA/Ch ratio and impregnation in the blend polymer scaffold formation. The voxel resolution was set to 11.49 µm. Reconstruction and analysis of the acquired 3D images were performed using DragonFly version 2022.2 from Object Research Systems (ORS). First, a specific region of interest (ROI) representative of the entire scaffold was isolated to assess the effect of working parameters on the resulting morphologies. A segmentation of the ROI was then performed to distinguish the void fraction (pores and pore connections, i.e., throats) from the solid material. This step is critical prior to 3D modeling as it will affect subsequent analysis and structure plots. A workflow called “pore network modeling” (OpenPNM) [40] determined the number of vertices (pores) and edges (throats) in the ROI selected.

### 2.5. Scanning Electron Microscopy

The morphology of the pores on the impregnated scaffolds was studied by scanning electron microscopy (SEM). A Nova NanoSEM 450TM (Elecmi, Zaragoza, Spain) was used with an accelerating voltage of 30 kV. A cross-section of each polymer, previously treated with liquid nitrogen to achieve a cleaner cut of the section and coated with a 10 nm gold layer, was selected prior to analysis.

### 2.6. Total Loaded Impregnation

A method of UV–Vis spectrophotometry was applied to quantify the amount of olive leaf extract loaded into the scaffolds, using a Synergy™ HTX Multi-Modal Microplate Reader (Izasa, Madrid, Spain). To perform this, each impregnated scaffold (10 mg) was completely degraded using 2.5 mL of dimethyl sulfoxide, which is capable of dissolving the PLGA. Subsequently, the absorbance of the solutions was measured at a detection wavelength of 660 nm. This wavelength represents the peak absorbance of OLE, without any interference from the chitosan or PLGA. To determine the final OLE impregnation percentage, a calibration curve was constructed in dimethyl sulfoxide. The impregnated load was determined using the following Equation (1):

(1) $$\%\mathrm{OLE}\;\mathrm{impregnated}=\frac{\mathrm{mOLE}}{\mathrm{mPolymers}}\times 100$$

Here, mOLE refers to the amount of ethanolic OLE impregnated into the polymers, while mPolymers represents the quantity of polymer. The impregnation loadings were measured in duplicate for accuracy. The total phenolic content (TPC) of the obtained foams was determined using the Folin–Ciocâlteu method as described by Singleton et al. with adaptations for use in microplates [41]. To measure the phenolic content, a calibration curve based on gallic acid as a standard was used, and the absorbance of each sample was measured at 725 nm. The obtained results were expressed in µg gallic acid equivalents per gram of polymer.

### 2.7. Antioxidant Capacity of Polymeric Scaffolds

The antioxidant capacity of polymeric scaffolds enriched with bioactive compounds from olive leaves was evaluated using the DPPH free radical spectroscopic method, based on a previously established protocol with some modifications [42]. First, the antioxidant capacity of the extract was evaluated at different concentrations using a 6 × 10^−5^ M ethanolic DPPH solution. Then, 7 µL of each concentration of OLE was combined with 293 µL of the DPPH solution and allowed to react for 2 h in the dark. After incubation, the absorbance was measured at 515 nm using the microplate reader. The percentage of oxidation inhibition (%OI) was then calculated using Equation (2):

(2) $$\%OI=\frac{A_{0}-A_{i}}{A_{0}}\times 100$$

In the equation, *A*_0_ is the initial absorbance, while *A_i_* is the absorbance measured after 2 h. For the impregnated scaffolds, 10 mg each was immersed in 4 mL of the same DPPH solution. After 8 h, during which time the scaffold compounds diffused into the solution, the absorbance was measured at 515 nm. The percentage of inhibition (%OI) for the impregnated scaffolds was then calculated using Equation (2). All experiments were performed in duplicate.

## 3. Results and Discussion

### 3.1. PLGA/Chitosan Morphological Analysis

PLGA/Ch scaffolds and PLGA/Ch loaded with olive pruning waste extracts were obtained in a one-step process of foaming and impregnation assisted by supercritical CO_2_ as described in Section 2.3. The study focuses on how the presence of different ratios of chitosan in the scaffold formation affects first the morphology and then the influence of the extract. For this purpose, the pressure (10 MPa), temperature (308 K), and decompression rate (10 MPa/min) were kept constant and only the chitosan content (50, 30, and 10 wt%) was varied. In the case of the foaming/impregnation process, the amount of extract used was always the same, 15 mL. It is important to note that the key criteria for optimal scaffolds are the presence of macropores (100–1000 μm) and robust interconnectivity [43].

All the data used for the analysis of porosity, pores, connections, and expansion factors were obtained using the DragonFly software on the X-ray tomography images. In the case of pores and connectivity, the workflow created by [40] for DragonFly was followed as a reference.

#### 3.1.1. Influence of Chitosan on Scaffold Formation

**Figure 3:**
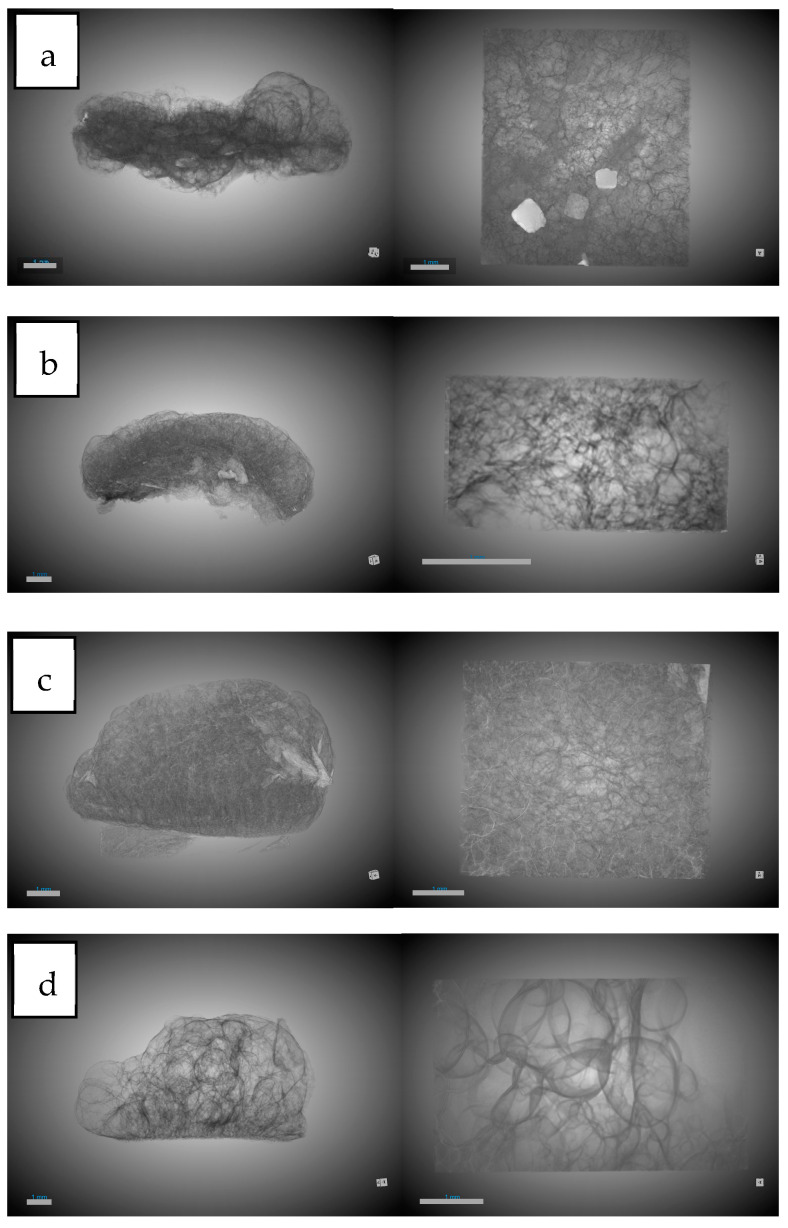
The 3D images of foaming disk and region of interest (ROI) selected for morphological analysis. (**a**) PLGA/Ch ratio 1:01; (**b**) PLGA/Ch ratio 7:03; (**c**) PLGA/CH ratio 9:1; (**d**) raw PLGA. Scale bar 1mm.

Table 3 shows the results of the analysis carried out for the main concepts of scaffold morphology. The first remarkable result was about expansion factor, which shows a decrease in the expansion factor as the amount of chitosan increases, from 91.11% for PLGA to 76.5% for PLGA/Ch with a ratio of 1:01. This can be explained by the lower amount of PLGA in the sample, which was the polymer capable of foaming in the supercritical conditions studied. Also, the presence of chitosan could break the biggest bubbles during the foaming process. The porosity seems to show a similar behavior to the expansion factor, but the effect of the presence of chitosan was not noticeable until the presence of 30 wt% chitosan was reached. PLGA and PLGA/Ch (9:01) have porosities of 85.55% and 88.55%, respectively, while PLGA/Ch (7:03) and PLGA/Ch (1:01) have porosities of 76.84% and 64.06%, respectively. In this case, Nie et al. [34] reported for PLGA/Ch formation under similar pressure conditions that the porosity increased slightly for weight ratios of 5 and 10 wt%. This is in line with a small increase in the porosity observed in this study for the (9:01) experiment, similar to the 10 wt% reported by Nie et al., but a significant compression was observed when the chitosan wt% exceeds this limit. In Figure 3, 3D images of the full disk after the foaming process and the region of interest (ROI) used to study the porosity are represented.

**Figure 4:**
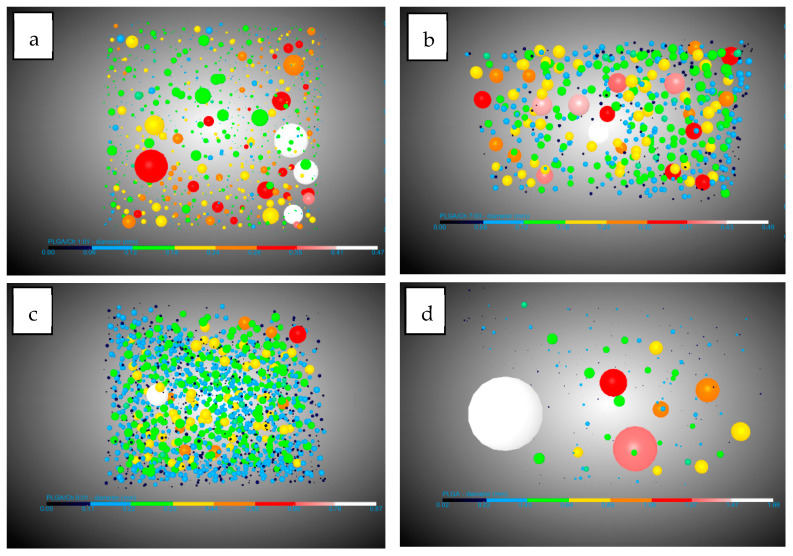
DragonFly images of PLGA/Ch: (**a**) 1:01, (**b**) 7:03, (**c**) 9:01, and (**d**) PLGA pore diameters.

**Figure 5:**
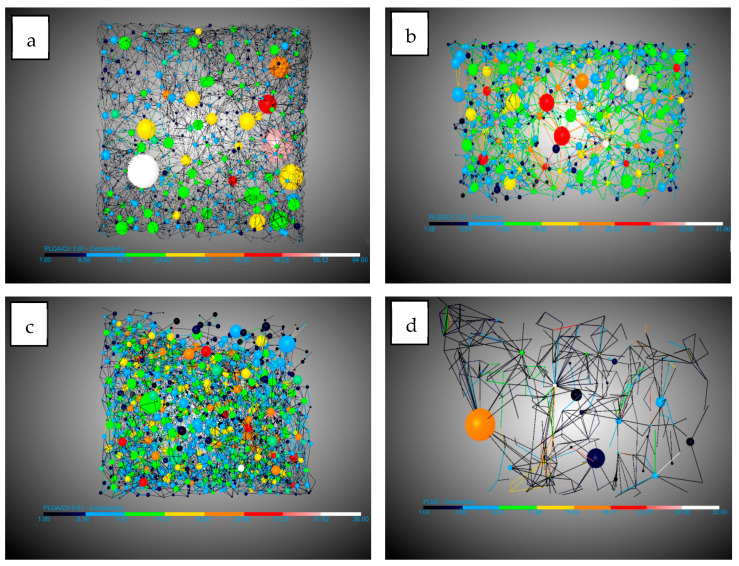
DragonFly images of PLGA/Ch: (**a**) 1:01, (**b**) 7:03, (**c**) 9:01, and (**d**) PLGA interconnectivity.

Looking at the pores, there are three important facts. The pore diameter (Figure 4) decreases in the presence of chitosan from 0.19 mm for PLGA to 0.11 mm in the two experiments with the highest amount of chitosan. This information should be handled carefully due to the high dispersion in the pore diameter shown by the SD. This could be due to the interference in nucleation caused by the difference in density of the two polymers: while PLGA has a density of 1.53 g/mL (Sigma Aldrich Product Reference), chitosan and derivatives have a density between 0.20 and 0.38 g/mL [44]. The number of pores/mm^3^ show a significant increase when chitosan was added compared to PLGA. In this case it goes from 8.73 pores/mm^3^ for PLGA to a maximum of 50.04 pores/mm^3^. It is worth noting that the maximum number of pores were found in the experiment where the amount of chitosan added was the lowest (9:01). This suggests that the presence of chitosan during foaming breaks up larger bubbles, creating smaller ones and promoting greater nucleation and pore formation. However, an excess of chitosan hinders the full development of PLGA during the foaming process. The final important pore data concern interconnectivity (Figure 5). The analysis shows that in all cases the presence of chitosan increases the average number of connections per pore by a factor of 2.5, with little difference between experiments carried out at different ratios. This is in agreement with the findings of Nie et al. [34] who indicate increased interconnectivity with 5 wt% chitosan and complete connectivity with 10 wt%, confirming the similarity of the connections for the chitosan ratios studied in this article.

**Figure 6:**
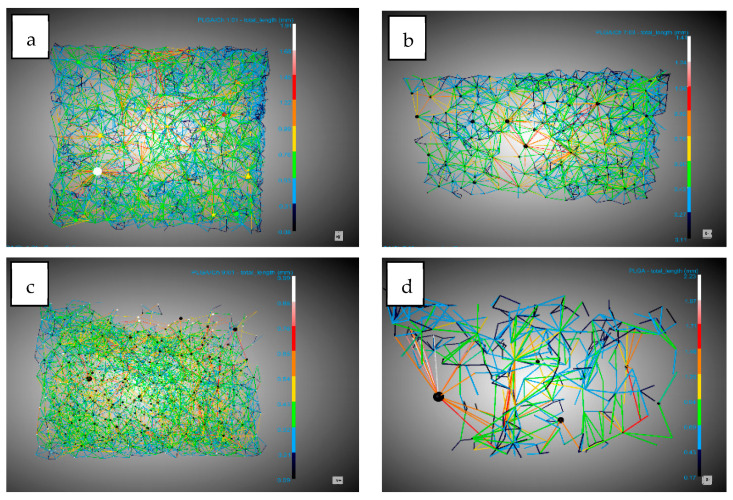
DragonFly images of PLGA/Ch: (**a**) 1:01, (**b**) 7:03, (**c**) 9:01, and (**d**) PLGA throats (conduits).

The other important concept in the study of morphology was that of conduits (throats) observed in Figure 6. In this study their numbers per mm^3^ were analyzed. The number of junctions per mm^3^ increased by 10 and 15 times compared to the number of junctions per mm^3^ in PLGA without the presence of chitosan. This is because a higher expansion factor leads to thinner walls, which are easier to break with chitosan increasing the number of pores and throats.

#### 3.1.2. Influence of Olive Pruning Extract on Scaffold Formation

**Figure 7:**
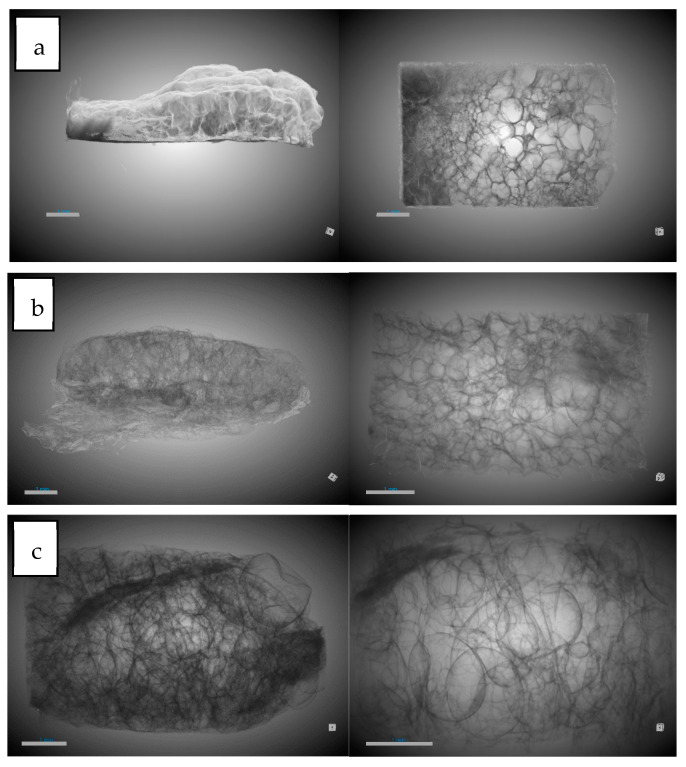
The 3D images of foaming disk and region of interest (ROI) selected for morphological analysis. (**a**) PLGA/Ch/olive extract ratio 1:01; (**b**) PLGA/Ch/olive extract ratio 7:03; (**c**) PLGA/CH/olive extract ratio 9:1. Scale bar 1 mm.

Table 4 shows the results of the tests carried out with the addition of olive pruning extract. In this case, a comparison was made with the morphology without the extract, since the literature does not show any impregnation of PLGA/Ch with a natural extract using supercritical processes. Similar to the experiments without extract, the expansion factor decreases when chitosan was added to the foaming process, but this expansion factor decreases significantly in the experiment with the highest amount of chitosan and extract (53.96%) compared to when only chitosan was added (76.5%). The porosity follows a similar pattern as in the absence of extract, but the decrease was attenuated by percentages ranging from 87.35–76.39% compared to experiments without extracts, which ranged from 88.55–64.06%. In Figure 7, 3D images of full disk after foaming and impregnation process and the region of interest (ROI) used to study the porosity are represented.

**Figure 8:**
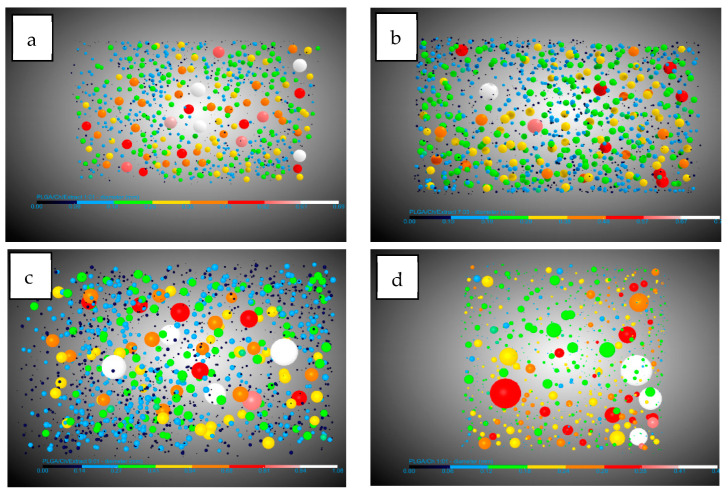
DragonFly images of PLGA/Ch/extract: (**a**) 1:01, (**b**) 7:03, (**c**) 9:01 and (**d**) PLGA pore diameter.

**Figure 9:**
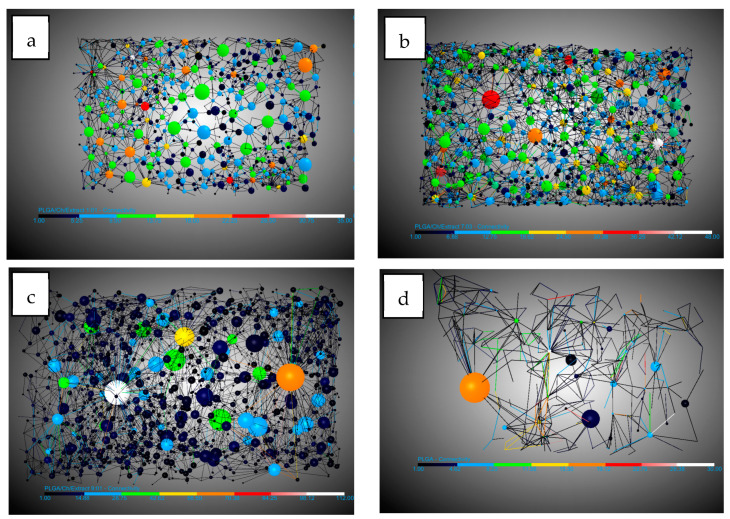
DragonFly images of PLGA/Ch/extract: (**a**) 1:01, (**b**) 7:03, (**c**) 9:01 and (**d**) PLGA interconnectivity.

With regard to the data on pores, a diameter reduction compared to PLGA was observed. This reduction in diameter was lower (0.13–0.14 mm) compared to the tests carried out without extract (with a minimum of 0.11 mm) (Figure 8). This information should be handled carefully due to the high dispersion in the pore diameter shown by the SD. The number of pores per mm^3^ observed were similar with respect to experiments carried out without extract except for the 9:01 ratio with a significant decrease in the presence of extract with 28.08 pores/mm^3^ compared to 50.04 pores/mm^3^ without extract. A notable difference was observed in the average number of connections per pore (Figure 9), which decreased in all cases. In the case of the 1:01 tests, there was a reduction from 8.02 to 4.95, in the case of the 7:03 tests the reduction was from 8.41 to 6.9, and in the case of the 9:1 tests the reduction due to the presence of the extract was from 7.72 to 5.38. This decrease can be explained by the presence of the extract in the ducts, which, being small, are filled and these connections between the pores are eliminated.

**Figure 10:**
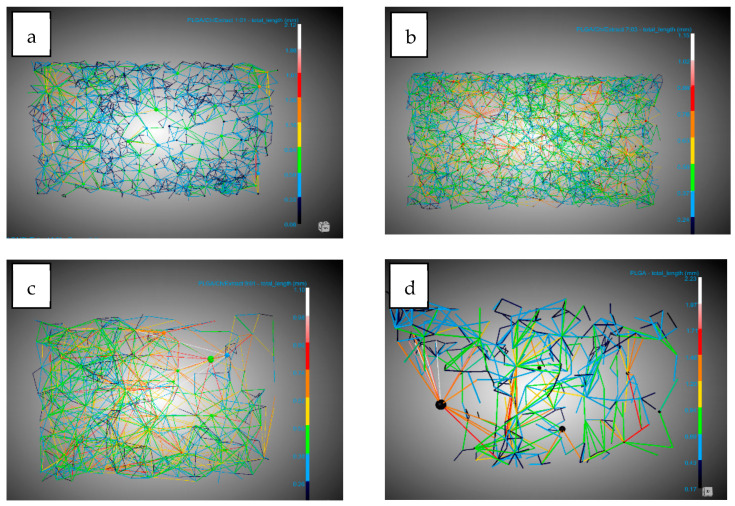
DragonFly images of PLGA/Ch/extract: (**a**) 1:01, (**b**) 7:03, (**c**) 9:01 and (**d**) PLGA throats (conduits).

Another piece of data that corroborates the previously mentioned presence of the extract was the decrease in the number of conduits per mm^3^ in these tests (Figure 10). In all cases, there was a significant decrease, with the greatest difference observed in the tests conducted with the 9:01 ratio, where it decreases from 193.35 conduits per mm^3^ to 73.27 conduits per mm^3^.

### 3.2. Extract Loading (%)

**Figure 11:**
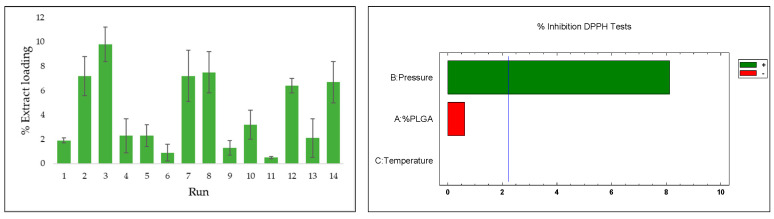
Load of %OLE on impregnated scaffolds and Pareto diagram showing the influence of the different variables used.

An olive leaf extract prepared using an improved extraction method was used in accordance with previous studies. In this case, the concentration of the OLE was set at 60 mg/mL in an ethanolic solution and an extraction efficiency of 18.3% was achieved at 200 bar and 80 °C for 3 h. The percentage of OLE loaded was evaluated in different experiments (shown in Table 2), while the effects of pressure, temperature, and the ratio of polymers were studied. The results observed under different parameters are shown in Figure 11.

The impregnation percentages of the extract were obtained within a range of 0.5–9.8%. Evaluating the impact or statistical importance of each of the parameters studied in the process (pressure, temperature, and ratio), only pressure showed a significant positive effect (*p*-value 0.0001) on the impregnation percentage of the extract in the produced scaffolds. The effect of the different variables can be seen in the Pareto diagram shown below. For pressure, it was clear that increased pressures led to greater impregnation of bioactive compounds. The optimal conditions (as explored in this study) for achieving a high percentage of OLE load impregnation were 30 MPa, 308 K, and 1:1 ratio for PLGA/chitosan.

Rising pressure triggers two key mechanisms that can increase impregnation levels in this type of process. Firstly, it alters the concentration gradient of polyphenols in the extract between the supercritical phase and the polymer, affecting the final impregnation result. In addition, the solubility of these compounds in CO_2_ increases with pressure, leading to saturation as the extract is over-concentrated in the impregnation vessel. This creates a concentration gradient of the target compounds, favoring their uptake into the polymer matrix. This effect for pressure is corroborated in several previous works [45,46]. Moreover, when combined with a parallel foaming process, the phenomenon of polymer expansion, which increases at higher pressures, can facilitate the uptake of compounds during impregnation by increasing diffusion through the polymer. The significant influence of pressure observed in the experiments carried out can also be attributed to this mechanism.

The key factor governing the incorporation of the extract is the affinity between the polymeric material, such as the PLGA–chitosan mixture, and the APIs. The study of potential interactions between the extract components and the polymer is challenging due to the complex composition of the extract. Nevertheless, the functionalities of the primary constituents are taken into account in the analysis. The ethanolic extracts of olive leaves obtained by supercritical CO_2_ extraction contain mainly oleuropein, various derivatives of luteolin, and hydroxytyrosol [47]. For these compounds to be incorporated into the polymer by supercritical impregnation, significant chemical interactions must allow molecular dispersion within the polymer. These major extract compounds have multiple hydroxyl groups that can form hydrogen bonds with the carbonyl groups in the PLGA chains [48] or with the chitosan, which may also cause plasticizing effects of the polymer [49]. In addition, the highly hydrophilic character of these polyphenols can influence the partition coefficient, favoring the polymer over the supercritical phase and affecting the impregnation process. The total polyphenol content (TPC) of the samples was analyzed and maximum values of 242.4 and 256.9 µg gallic acid/g of polymer were obtained for runs 7 and 14, respectively. Phenolic content results were consistent with loading, with higher phenolic content generally obtained when high pressure was used.

### 3.3. Scanning Electron Microscopy

**Figure 12:**
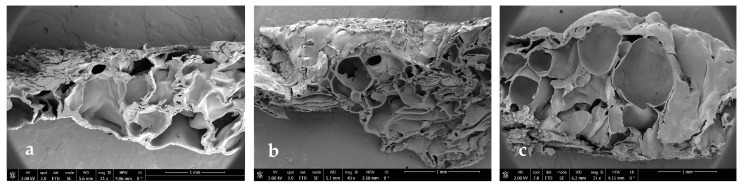
Microscopic image (SEM) of PLGA/Ch scaffolds after impregnation under conditions of P = 10 MPa, T = 308 K. (**a**) Ratio 1:01, (**b**) ratio 7:03, (**c**) ratio 9:01.

**Figure 13:**
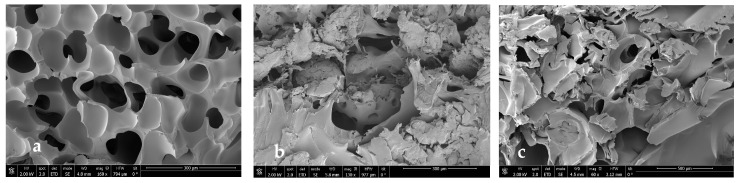
Microscopic image (SEM) of PLGA/Ch scaffolds after impregnation under conditions of P = 30 MPa, T = 328 K. (**a**) Ratio 1:01, (**b**) ratio 7:03, (**c**) ratio 9:01.

**Figure 14:**
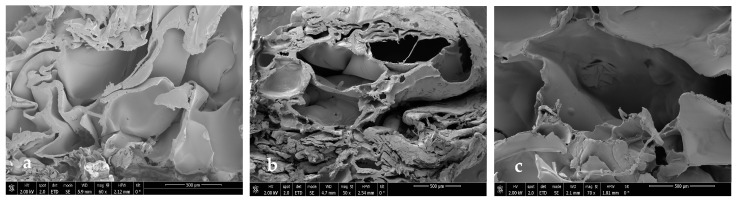
Microscopic image (SEM) of PLGA/Ch scaffolds after impregnation under conditions of P = 30 MPa, T = 308 K. (**a**) Ratio 1:01, (**b**) ratio 7:03, (**c**) ratio 9:01.

**Figure 15:**
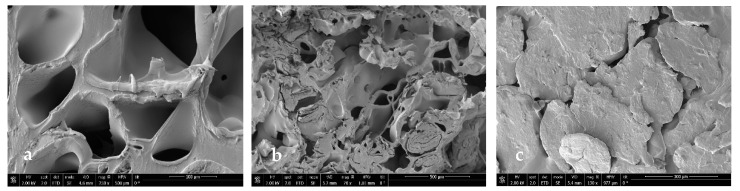
Microscopic image (SEM) of PLGA/Ch scaffolds after impregnation under conditions of P = 10 MPa, T = 328 K. (**a**) Ratio 1:01, (**b**) ratio 7:03, (**c**) ratio 9:01.

In order to see if part of the extract was impregnated and to see the internal morphology of the samples in more detail, SEM images of the impregnated scaffolds were taken under different conditions of pressure, temperature, and polymer ratio (Figure 12, Figure 13, Figure 14 and Figure 15).

In general, foams produced with a higher PLGA ratio clearly showed a more compact structure with better voids, while those prepared with a 1:1 ratio of PLGA/chitosan have much larger voids throughout the structure. The aforementioned mixing of micropores and mesopores can also be observed in the impregnated samples, which opens up a multitude of applications in tissue engineering. On the other hand, the active compounds present in the extract cannot be differentiated by SEM technology.

### 3.4. Antioxidant Activity

**Figure 16:**
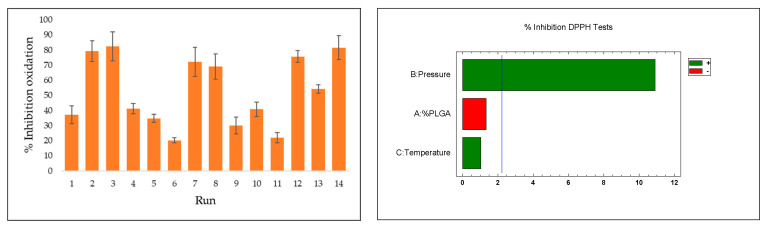
Percentages of oxidation inhibition for OLE-impregnated polymeric scaffolds and Pareto diagram showing the influence of the different variables used.

The antioxidant capacity of the impregnated scaffolds was measured using the DPPH test. The method can effectively evaluate the capability of compounds in olive leaf extract to neutralize DPPH and diminish free radicals. This property has been validated before both in its extract form and when incorporated into other matrices, showing significant levels of oxidation inhibition [28]. The accumulation of free radicals or reactive oxygen species (ROS) can result in a range of health issues, contributing to oxidative stress and potentially leading to cardiovascular or neurodegenerative disorders. Figure 16 presents the results for the scaffolds, indicated by the percentage of oxidation inhibition. To evaluate the impregnation efficiency and activity of the compounds, the extract was analyzed separately in its initial liquid form. The isolated extract showed a DPPH oxidation inhibition rate of 95.6%.

Overall, the findings align with those observed in the total impregnated load, with scaffolds produced at higher pressures (run 2: 79.4%; run 3: 82.5%; run 7: 72.3; run 8: 69.1%; run 12: 75.8%; run 14 81.7%) consistently showing greater inhibition. This variable is the key factor governing the impregnation–foaming process in this study, and pressure showed a significant positive effect (*p*-value 0.0001) on the inhibition of DPPH oxidation, whereas temperature or polymer ratio did not show significant results when switching between the different levels. Analogous results to those obtained in this work exist in the literature with respect to impregnation and antioxidant activity of natural extracts at high pressure. Using mango extract, DPPH inhibitions of around 80% were obtained in calcium alginate wound dressings at 30 MPa [50]. Higher antioxidant activity was also obtained using mango leaf extract on PLA filaments at 40 MPa [51].

## 4. Conclusions

The proposed process of forming porous polymeric PLGA scaffolds together with different ratios of chitosan in combination with the impregnation of an extract of olive pruning residues proved successful. The morphological characteristics of the scaffolds were studied in the presence of different ratios of chitosan. The expansion factor decreased as the ratio of chitosan present increased. This is also produced when the OLE was present. Variable pore diameters were obtained, decreasing in the presence of chitosan from 0.19 mm for only PLGA to 0.11 mm in the two experiments with the highest amount of chitosan. The interconnectivity of the resulting foams was studied and in most cases the presence of chitosan increased the average number of connections by a factor of 2.5. The effect of some key process variables in the supercritical impregnation was also investigated. Higher pressures favored the OLE loading by increasing the concentration gradient in excess and the diffusivity of CO_2_ within the polymer matrix. The optimal conditions for impregnation of the OLE were 30 MPa, 308 K, and 1:1 ratio for PLGA/chitosan. Scaffolds were tested for antioxidant activity, showing high inhibition of oxidation (82.5% in the best conditions) with significant potential against pathologies induced by oxidative stress. The combination of interconnectivity and antioxidant activity, along with varying porosity levels, supports the potential of using scaffolds produced by the proposed single-step foaming and impregnation method in applications such as bone, cardiovascular, osteogenesis, or neural tissue engineering. Additionally, future in vitro and in vivo tests are crucial to validate cell viability outcomes.

## Figures and Tables

**Table 1:** Design of experiments for foaming and foaming–impregnation process to study the morphology of scaffolds.

Fixed Parameters		Variable	
Pressure	10 MPa	PLGA/Ch Ratio	1:01, 7:03, 9:01
Temperature	308 K	Olive Pruning Extract	15 mL
CO_2_ Flow Rate	20 mg/min		

**Table 2:** Experimental design of mixed levels (3 × 2 × 2) with two center points to study the influence of T, P, and ratio in the impregnation process.

Runs	P (MPa)	T (K)	Ratio
1	10	308	1:1
2	30	328	7:3
3	30	308	1:1
4	10	328	9:1
5	10	328	7:3
6	10	308	7:3
7	30	328	9:1
8	30	308	9:1
9	10	328	1:1
10	20	318	7:3
11	10	308	9:1
12	30	308	7:3
13	20	318	7:3
14	30	328	1:1

**Table 3:** Microporous analysis results for the experiments carried out without extract.

Experiments	Ratio (PLGA/Ch)	Expansion Factor (Ƹ)	Porosity (%)	Pore Diameter and SD (mm)	Number of Pores/mm^3^	Connections per Pore and SD	Number of Throats/mm^3^
PLGA/Ch	1:01	76.50	64.06	0.11 ± 0.08	34.87	8.02 ± 4.30	133.04
PLGA/Ch	7:03	84.99	76.84	0.11 ± 0.08	46.32	8.41 ± 4.02	196.07
PLGA/Ch	9:01	89.67	88.55	0.15 ± 0.11	50.04	7.72 ± 2.95	193.35
PLGA	–	91.11	85.55	0.19 ± 0.14	8.37	3.26 ± 1.52	13.25

**Table 4:** Microporous analysis results for the experiments carried out with extract.

Experiments	Ratio (PLGA/Ch)	Expansion Factor (Ƹ)	Porosity (%)	Pore Diameter (mm)	Number of Pores/mm^3^	Connection per Pore	Number of Throats/mm^3^
PLGA/Ch	1:01	53.96	76.39	0.13 ±0.09	36.54	4.95 ± 2.24	90.86
PLGA/Ch	7:03	82.08	82.38	0.13 ± 0.09	47.41	6.90 ± 3.10	162.84
PLGA/Ch	9:01	88.54	87.35	0.14 ± 0.11	28.08	5.38 ± 3.38	73.27
PLGA	–	91.11	85.55	0.19 ± 0.14	8.37	3.26 ± 1.52	13.25

## Data Availability

Data are contained within the article.
